# Use of Antimicrobial Films and Edible Coatings Incorporating Chemical and Biological Preservatives to Control Growth of *Listeria monocytogenes* on Cold Smoked Salmon

**DOI:** 10.1155/2014/534915

**Published:** 2014-06-25

**Authors:** Hudaa Neetoo, Fawzi Mahomoodally

**Affiliations:** ^1^Department of Agriculture and Food Science, Faculty of Agriculture, University of Mauritius, Mauritius; ^2^Department of Health Sciences, Faculty of Science, University of Mauritius, Mauritius

## Abstract

The relatively high incidence of *Listeria monocytogenes* in cold smoked salmon (CSS) is of concern as it is a refrigerated processed food of extended durability (REPFED). The objectives of this study were to compare and optimize the antimicrobial effectiveness of films and coatings incorporating nisin (Nis) and sodium lactate (SL), sodium diacetate (SD), potassium sorbate (PS), and/or sodium benzoate (SB) in binary or ternary combinations on CSS. Surface treatments incorporating Nis (25000 IU/mL) in combination with PS (0.3%) and SB (0.1%) had the highest inhibitory activity, reducing the population of *L. monocytogenes* by a maximum of 3.3 log CFU/cm^2^ (films) and 2.9 log CFU/cm^2^ (coatings) relative to control samples after 10 days of storage at 21°C. During refrigerated storage, coatings were more effective in inhibiting growth of *L. monocytogenes* than their film counterparts. Cellulose-based coatings incorporating Nis, PS, and SB reduced the population of *L. monocytogenes*, and anaerobic and aerobic spoilage flora by a maximum of 4.2, 4.8, and 4.9 log CFU/cm^2^, respectively, after 4 weeks of refrigerated storage. This study highlights the effectiveness of cellulose-based edible coatings incorporating generally regarded as safe (GRAS) natural and chemical antimicrobials to inhibit the development of *L. monocytogenes* and spoilage microflora thus enhancing the safety and quality of CSS.

## 1. Introduction

*Listeria monocytogenes* has long been established as an important food-borne pathogen with a fatality rate of 25–30% [1]. However, the incidence of food-related listeriosis has increased dramatically in the last few years, where* L. monocytogenes* has been listed in the top five highest-ranking pathogens with respect to the total cost of foodborne illness in the United States in terms of loss of income by the affected individual, cost of health care, loss of productivity due to absenteeism, costs of investigations of an outbreak, loss of income due to closure of businesses, consumer litigations, or losses of product sales when consumers avoid particular products [2].

*L. monocytogenes* infection has been associated with consuming a variety of meat, poultry, fish, and dairy products [3]. The prevalence of this organism in cold smoked fish in particular is relatively high and typically between 10 to 40% [4, 5]. This high prevalence is likely due to the low temperature inherent with cold smoking process. Indeed, this condition would be congenial for the proliferation of* L. monocytogenes* if the raw salmon harbored the pathogen or acquired it from the processing environment [6]. Hence, processing of cold smoked salmon (CSS) includes no recognizable critical control point for* L. monocytogenes* and therefore this product cannot be completely free of this pathogen [7]. For this reason, smoked seafood, including CSS, has been categorized as having a high risk of listeriosis [7].

Since postprocess contamination of smoked fish with* L. monocytogenes* is highly problematic, antimicrobial additives are sought after to prevent growth of this bacterium in food products and to ensure safety. Several studies have reported the effectiveness of nisin (a polycyclic antibacterial peptide) in delaying and reducing growth of* Listeria spp.* in model systems [8, 9] and in RTE products [10] including CSS [11, 12]. Nisin is a heat-stable bacteriocin that kills sensitive pathogens by disrupting their cell membranes, leading to leakage of cellular material and ultimately cell death [13]. In the United States, nisin has a generally regarded as safe (GRAS) status for use in pasteurized processed cheese and petitions have been filed for its use in other products as well [14, 15]. The efficacy of nisin as an antimicrobial agent in raw or minimally processed seafood is limited by several factors [16]. These include the potential for proteolytic activity in raw food that would cause rapid degradation of nisin [17], as well as the rapid decrease in the antimicrobial activity of nisin due to increased resistance of the pathogens [18].

Thus, a multiple-hurdle approach relying on the combination of nisin with other antimicrobials, such as chemical preservatives, is desirable. Sodium lactate (SL) is a GRAS additive that is widely used to enhance flavor, control microbial growth, and increase shelf life of meat, poultry, and fish products [19–22]. The use of lactates as antimicrobial agents is primarily due to their ability to reduce pH and water activity. Currently, the addition of SL is allowed at 4.8% for the decontamination of seafood products [16]. Sodium diacetate (SD), a derivative of acetic acid, is used to achieve an antimicrobial effect in baked goods, fats and oils, gravies and sauces, snack foods, meat products, and soups and soup mixes, as well as to flavor these foods [16]. It is also a GRAS substance recommended for use at levels not exceeding 0.25% [16]. Potassium sorbate (PS) is a salt of sorbic acid and common usage levels of PS in various food products have ranged from 0.5 to 1.0% [23]. Depending on the processing conditions, PS is usually applied to whole or eviscerated fish or fillets, prior to or immediately after smoking [24]. Sorbates may be used at a level not exceeding 0.3%, and at this concentration sorbates do not contribute to flavor [23]. Sodium benzoate (SB) is also used as an antimicrobial agent and is currently allowed at 0.1% [16]. These chemical preservatives have been shown to inhibit growth of gram-positive bacterial pathogens such as* L. monocytogenes* in media, meat [25–30], and seafood [31, 32].

Antimicrobial coatings and films allow the controlled diffusion and gradual release of embedded antimicrobials onto the food surface [33]. Significant inroads have been made in antimicrobial packaging to control the proliferation of* L. monocytogenes* on CSS. Neetoo et al. [34] found that alginate coatings supplemented with 2.4% SL and 0.25% SD significantly delayed the growth of* L. monocytogenes* in CSS during a 30-day storage at 4°C. Moreover, Ye et al. [35] showed that prior frozen storage enhanced the effect of alginate-based coatings and chitosan-based films incorporated with SL (1.2 or 2.4% w/w) or SD (0.125 or 0.25% w/w) against* L. monocytogenes* on CSS during subsequent refrigerated storage. Reductions ranging from 0.5 to 4.5 log CFU/cm^2^ compared to uncoated samples were reported. Growth of* L. monocytogenes* on the surface of CSS was inhibited by whey-protein films incorporating a lactoperoxidase system [36]. Seydim and Sarikus [37] showed that strong antimicrobial activity of oregano essential oil impregnated in whey-protein isolate-based edible films against* L. monocytogenes*. Similarly, Tammineni et al. [38] developed an edible antimicrobial film using potato peel waste incorporating oregano essential oil against* L. monocytogenes* on CSS. The films reduced the inoculum by greater than 2 log cfu/g during storage at 4°C for 28 days. However, all aforementioned studies have tested the antilisterial efficacy of films or coatings in isolation and lack a systematic comparison of the relative efficacy of each approach.

The objective of this study was to compare cellulose-coated plastic films and edible cellulose-based coatings incorporating binary or ternary combinations of food-approved antimicrobials to inhibit growth of* L. monocytogenes* and spoilage microflora on vacuum-packaged CSS during refrigerated storage (4°C).

## 2. Materials and Methods

### 2.1. *Listeria monocytogenes* Inoculum Preparation

Five* L. monocytogenes* strains, PSU1 (serotype 1/2a), PSU21 (serotype 4b), PSU9 (serotype 1/2b), F5069 (serotype 4b), and Scott A (serotype 4b) (Courtesy of Rolf Joerger, University of Delaware), were used. The strains were maintained on tryptic soy agar plus 0.6% yeast extract (TSAYE) plates and stored at 4°C. Each strain was grown independently in tryptic soy broth plus 0.6% yeast extract (TSBYE) for 24 h at 37°C and a loopful of each overnight culture was transferred to 10 mL of fresh TSBYE and incubated at 37°C for 24 h. On the day of the experiment, 1 mL volume of each culture was combined to provide a five-strain mixture and then readjusted with 0.1% peptone water to a final cell density of ca. 10^8^ CFU/mL, which served as the inoculum. Serial dilutions were plated onto TSAYE plates and incubated at 37°C for 24 h to determine cell numbers.

### 2.2. Inoculation of CSS Samples

Freshly processed CSS (*Salmo salar*) was obtained from a producer. It was kept frozen at −20°C and thawed at 2 ± 2°C (<4°C) for 1 day immediately before use. Slices of CSS were punched aseptically into 5.7 cm diameter round pieces weighing 10 ± 1 g. The samples were surface-inoculated with a 10^8^ CFU/mL dilution of the five-strain cocktail of* L. monocytogenes* to achieve final concentrations of 10^5^ CFU/cm^2^ (or 5 × 10^5^ CFU/g) of salmon surface. After inoculation, salmon samples were kept at room temperature for 30 min to allow bacterial attachment.* L. monocytogenes* populations in CSS are generally low (1–10^3^ CFU/g) with 90–99% of cases below 10^2^ CFU/g and less than 1% between 10^3^ and 10^4^ CFU/g as reported by Jørgensen and Huss [39] and Farber and Peterkin [40]. However, pathogen levels as high as 10^5^–10^7^ CFU/g have also been reported previously [1]. In this experiment, we used an initial load of ~5 log CFU/g to provide a worst-case scenario. From a risk assessment point of view, using an inoculum size of 5 log CFU/g to test the antilisterial efficacy of antimicrobial films and coatings provides greater confidence or assurance.

### 2.3. Antilisterial Effectiveness of Films and Coatings Incorporating Binary Combinations of Antimicrobials

#### 2.3.1. Preparation of Coating Solution

Methylcellulose (MC; 7.0 g) and hydroxypropylmethylcellulose (HPMC; 3.0 g) were mixed with 200 mL of 95% ethanol and 200 mL of sterile distilled water and stirred to which 6 mL of polyethylene glycol 400 was subsequently added. This coating stock solution was then supplemented with the various antimicrobial preparations. Briefly, 1.3 g of nisin was dissolved in 60 mL of 0.02 M acetic acid, and 12 mL of the prepared nisin solution was then supplemented with either 1.29 g SD, 1.54 g PS, 0.51 g SB, or 2.57 g of a-60% SL syrup. The antimicrobial solution was then made up to 50 mL with the MC/HPMC carrier solution. The solution was sufficient to coat 10 salmon discs.

#### 2.3.2. Preparation of Films

The MC and HPMC were mixed with ethanol and water to prepare a coating solution as described above. The coating solution was then supplemented with the various antimicrobials. Briefly, 3.0 g nisin was dissolved in 60 mL 0.02 M acetic acid, and 12 mL of the prepared nisin solution was then supplemented with 3.0 g SD, 3.6 g PS, 1.2 g SB, or 6 g of 60% of SL syrup. The antimicrobial solution was then made up to 42 mL with the MC/HPMC carrier solution. The solution was then used to coat 3 glass plates, lined with LDPE films covering a surface area of 400 cm^2^ each, using a thin layer chromatography (TLC) plate coater.

#### 2.3.3. Treatment of Inoculated CSS Samples with Antimicrobial Coatings and Films

The inoculated CSS discs were coated with a 500 *μ*L aliquot of a HPMC/MC coating solution containing nisin (25,000 IU/mL) alone or in combination with SL (0.3%), SD (0.25%), PS (0.3%), or SB (0.1%) as summarized in Table 1. The samples were then air-dried by leaving them in a laminar-flow hood under ventilation for 20 min. Samples were then flipped and similarly coated with an equal volume of the antimicrobial coating solution followed by drying. Alternatively, inoculated CSS discs were wrapped in different antimicrobial-coated films as shown in Table 1.

Inoculated samples without films or coatings were also prepared as untreated controls. Controls and treated samples (film-wrapped or coated) were then inserted into 3 mm thick high barrier pouches and subsequently sealed using a vacuum-packaging machine. The samples were stored at room temperature (21°C) for 10 days and analyzed microbiologically every other day.

#### 2.3.4. Enumeration of* L. monocytogenes* from Samples

For microbial analysis, the package was aseptically cut and the sample transferred to a sterile stomacher bag that contained 40 mL of 0.1% sterile peptone water and stomached for 2 min. Serial dilutions were made in 0.1% peptone water and counts of* L. monocytogenes* were determined by an overlay method [41]. Briefly, the serial dilutions were spread-plated on solidified TSAYE and the plates were incubated at 35°C for 3 h. where Modified Oxford Medium (~7 mL) tempered at 45°C was overlaid on the plates. These plates were then incubated at 35°C for 48 h and small black colonies with black haloes were counted. Occasionally, suspect colonies were confirmed using a BAX for Screening/*Listeria monocytogenes* PCR assay. The numbers of* L. monocytogenes* per cm^2^ of salmon were calculated by dividing the total count of* L. monocytogenes* per salmon disc by the total surface area (51.4 cm^2^). The absence of the pathogen in the CSS samples was confirmed by a primary enrichment in UVM broth (Difco Laboratories) and a secondary enrichment in Fraser broth (Difco Laboratories) according to the USDA Microbiology Laboratory Guidebook [42].

### 2.4. Antilisterial Effectiveness of Films and Coatings Incorporating Ternary Combinations of Antimicrobials

The CSS samples were inoculated to a final concentration of 10^5^ CFU/cm^2^ of salmon surface. Antimicrobials with the highest antilisterial activity (PS and SB) were selected for further testing. Inoculated salmon discs were coated with a 500 *μ*L aliquot of HPMC/MC coating solution on each side containing Nis (25000 IU/mL) with PS (0, 0.15 or 0.3%) or Nis (25000 IU/mL) with PS (0, 0.15, or 0.3%) and SB (0, 0.05,or 0.1%). The samples were air-dried and packaged. In addition, inoculated CSS samples were wrapped with LDPE films coated with a solution containing Nis (25000 IU/mL) with PS (0, 0.15, or 0.3%) or Nis (25000 IU/mL) with PS (0, 0.15, or 0.3%) and SB (0, 0.05, or 0.1%). Samples were then packaged and stored at 21°C for 10 days. The formulations for the various binary or ternary combinations of antimicrobials incorporated in films or coatings are summarized in Table 2. Samples were then microbiologically analyzed for* L. monocytogenes* as described earlier.

### 2.5. Effectiveness of Films and Coatings Containing Selected Antimicrobial Combinations against* L. monocytogenes* and Spoilage Microflora

The CSS samples were surface-inoculated to a final concentration of approximately 10^3^ CFU/cm^2^ (or 5 × 10^3^ CFU/g). Uninoculated samples were also prepared. Inoculated and uninoculated samples were wrapped with film or coated with an antimicrobial coating solution, packaged, and stored at 4°C for 4 weeks. The combinations of antimicrobials incorporated in films and coatings for the refrigerated storage study are summarized in Table 3.

Inoculated samples were analyzed weekly for* L. monocytogenes* as described previously. Uninoculated samples were also analyzed weekly for spoilage aerobic and anaerobic bacteria. Spoilage anaerobic bacterial counts were determined by plating on Liver Veal Agar and plates incubated in anaerobic jars with anaerobic GasPak (BBL) for two days at 35°C. Aerobic bacterial counts were determined by plating onto TSAYE and plates incubated aerobically for two days at 35°C.

### 2.6. Statistical Analysis

Three independent trials were conducted for all experiments. Colony counts were converted to log_10_ CFU/cm^2^ and means and standard deviations were calculated using Microsoft Excel. A Tukey-Kramer test was used to determine differences in the populations of* L. monocytogenes*, aerobes, and anaerobes on CSS samples. Significant differences were considered at the 95% confidence level (*P* < 0.05).

## 3. Results

### 3.1. Antilisterial Effectiveness of Films and Coatings Incorporating Binary Combinations of Antimicrobials

**Figure 1:**
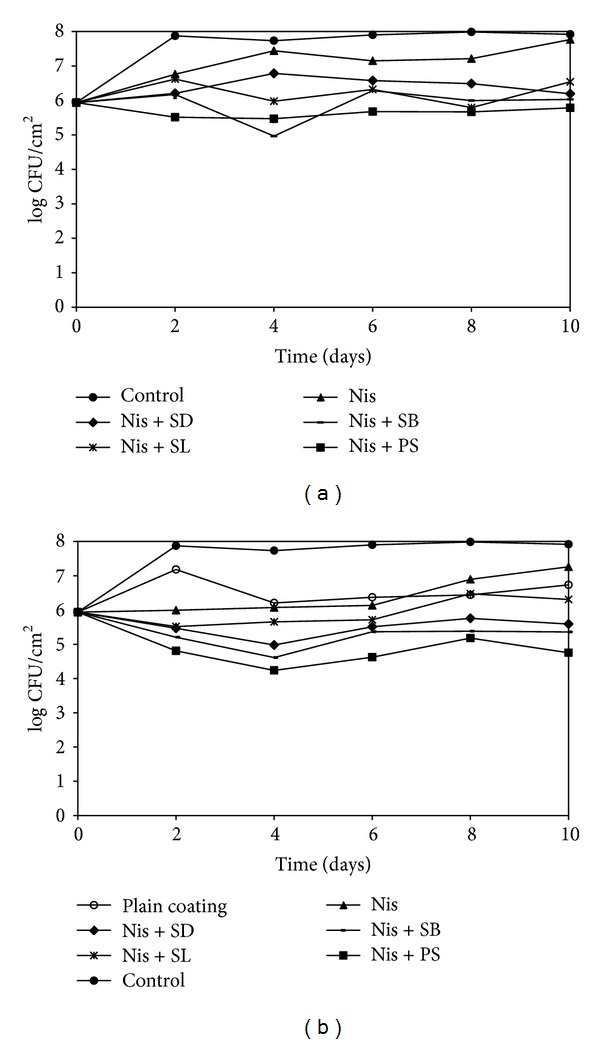
Fate of* L. monocytogenes* on CSS slices packaged with antimicrobial films (a) and coatings (b) incorporating nisin (25000 IU/mL), SL (0.3%), SD (0.25%), SB (0.1%), and PS (0.3%) and stored at ambient temperature. Error bars are omitted from the chart for the sake of clarity.

The fate of* L. monocytogenes* on CSS slices treated with different binary combinations of antimicrobials in films and coatings is represented in Figures 1(a) and 1(b), respectively. The initial load of the inoculum was about 6 log CFU/cm^2^. After 2 days, the control (untreated) samples had higher counts than all other treatments with the Nis + SB (5.2 log CFU/cm^2^) and Nis + PS (4.8 log CFU/cm^2^) coatings showing significantly (*P* < 0.05) lower counts than the control groups. After 10 days,* L. monocytogenes* population on samples treated with Nis + SB (4.9–6.3 log CFU/cm^2^) and Nis + PS (4.3–4.7 log CFU/cm^2^) was appreciably lower compared to the control untreated samples (7.7 log CFU/cm^2^) although the results were not statistically significant (*P* > 0.05).

### 3.2. Antilisterial Effectiveness of Films and Coatings Incorporating Ternary Combinations of Antimicrobials

**Figure 2:**
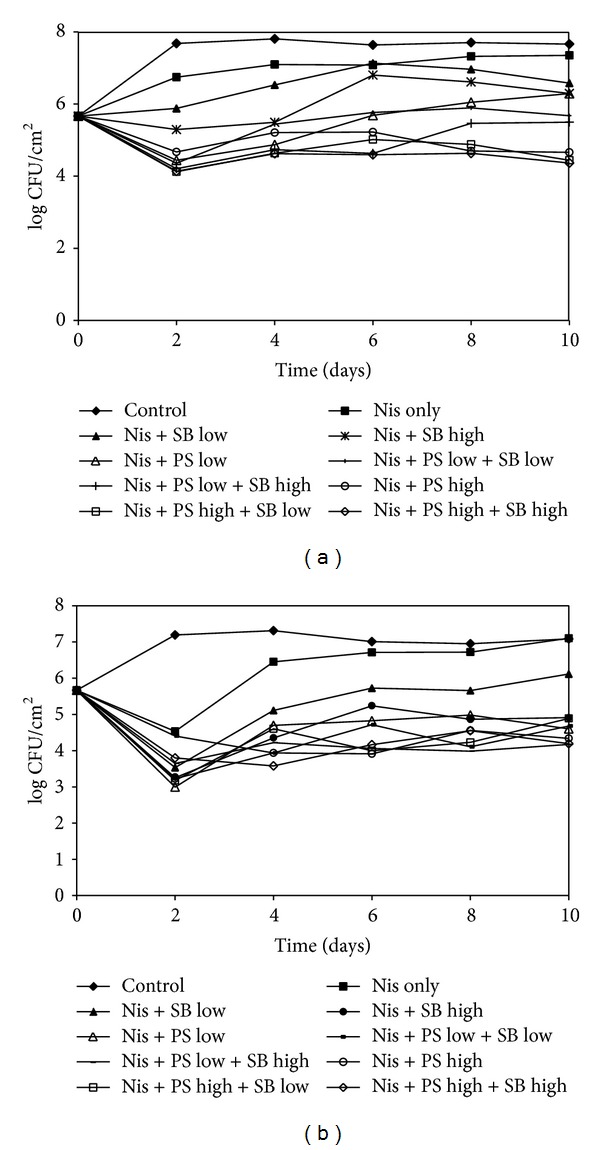
Populations of* L. monocytogenes* on CSS slices packaged with antimicrobial films (a) and coatings (b) incorporating nisin (25000 IU/mL) with PS at low (0.15%) or high (0.3%) concentrations or SB at low (0.05%) or high concentrations (0.1%) and stored at ambient temperature. Error bars are omitted from the chart for the sake of clarity.

Figures 2(a) and 2(b) show the effect of film and coating treatments incorporating ternary combinations of antimicrobials on the growth of* L. monocytogenes* on CSS. The initial counts of* L. monocytogenes* on CSS were about 5.6 log CFU/cm^2^ and increased steadily over the 10-day period reaching a maximum count of 7.6 log CFU/cm^2^. Over the storage period, the counts for all treatments were consistently lower than the control for either method of antimicrobial application. Ternary combinations of Nis (25000 IU/mL), PS (0.15 or 0.3%), and SB (0.05 or 0.1%) in films and coatings significantly (*P* < 0.05) inhibited the growth of* L. monocytogenes* after 10 days, lowering the population by 2.0–3.3 log CFU/cm^2^ and 2.2–2.9 log CFU/cm^2^_,_ respectively.

### 3.3. Effectiveness of Films and Coatings Containing Selected Antimicrobial Combinations against* L. monocytogenes* and Spoilage Microflora

**Figure 3:**
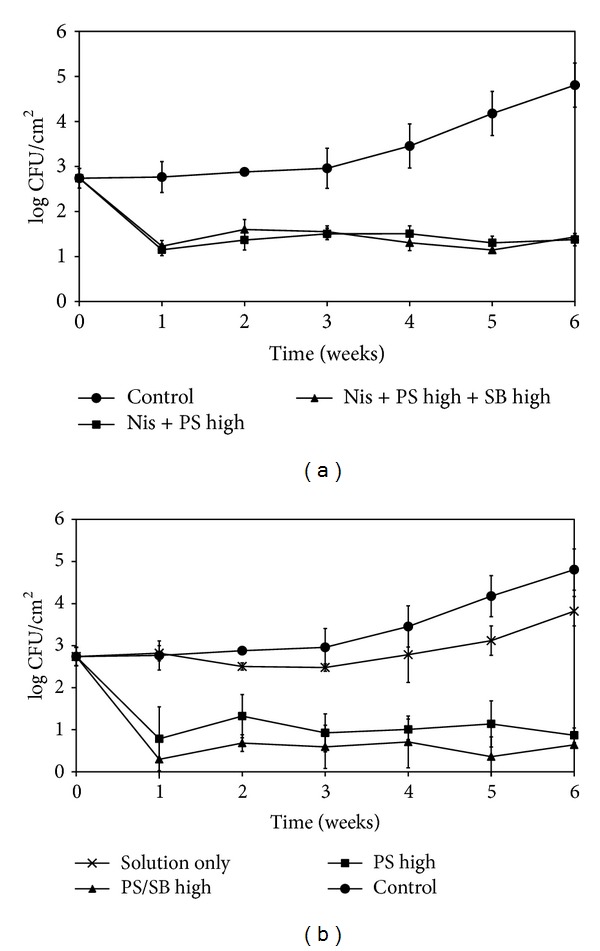
Development of* Listeria monocytogenes* on CSS slices treated with antimicrobial films (a) and coatings (b) incorporating Nis (25000 IU/mL) with high concentrations of PS (0.3%) with or without SB (0.1%) during storage at 4°C.

The two combined treatments chosen for this study were Nis + PS (0.3%) and Nis + PS (0.3%) + SB (0.1%).* L. monocytogenes* counts on inoculated CSS treated with antimicrobial films and coatings incorporating Nis + PS or Nis + PS + SB are shown in Figures 3(a) and 3(b), respectively. The mean population of* L. monocytogenes* on CSS recovered just after inoculation was 2.7 log CFU/cm^2^. The pathogen grew unhindered in untreated samples stored at 4°C, reaching approximately 4.8 log CFU/cm^2^ after 4 weeks. All the antimicrobial treatments (films or coatings) brought about a significant reduction (*P* < 0.05) in the population achieving reductions of 2.0–2.7 log CFU/cm^2^ by the end of the storage period. Ternary combinations of Nis (25000 IU/mL) with PS (0.3%) and SB (0.1%) were more effective than binary combinations of Nis (25000 IU/mL) with PS (0.3%) for either method of application although the difference was not statistically significant (*P* > 0.05).

**Figure 4:**
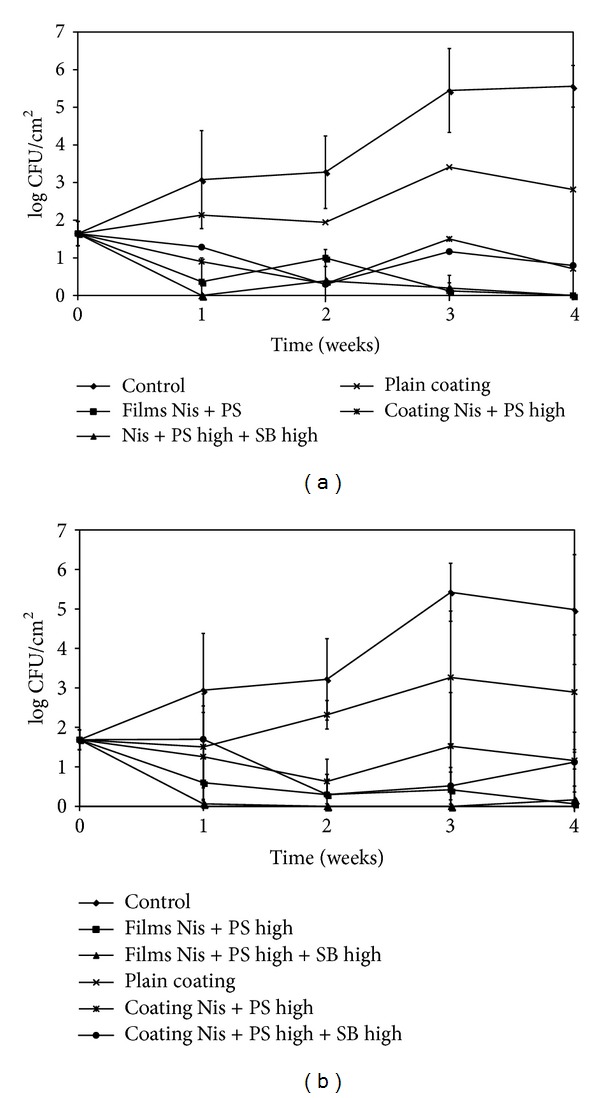
Development of mesophilic anaerobes (a) and aerobes (b)on CSS slices treated with antimicrobial films and coatings incorporating Nis (25000 IU/mL) with high concentrations of PS (0.3%) with or without SB (0.1%) during storage at 4°C.

The total anaerobic and aerobic counts of the uninoculated samples are shown in Figures 4(a) and 4(b). Throughout the storage study, the population of mesophilic aerobic and anaerobic bacteria in treated samples was consistently lower than their untreated counterpart with a maximum reduction of 4.9–5.6 log CFU/cm^2^ by the end of the storage period. Although both films and coatings were effective in delaying the development of background flora, coatings incorporating Nis + PS and Nis + PS + SB resulted in greater (*P* > 0.05) population reduction than their film counterparts.

## 4. Discussion

Cold smoked salmon (CSS) is considered a high-risk food because the temperature used during the cold smoking operation is not lethal to* L. monocytogenes*. In addition, CSS is a type of minimally processed food, also called Refrigerated Processed Foods of Extended Durability (REPFED) [43], and concern has been expressed about the survival and growth of this pathogen during the product's prolonged shelf life. During the past decade, there have been several recalls of smoked fish because of* L. monocytogenes* contamination [44] and it has been generally assumed that the presence of the pathogen on fish products is the result of postprocess contamination on the surface of the product [44]. Moreover, several studies [45, 46] have reported that bacterial population in cold smoked salmon can increase by 3-4 log CFU/g in a few weeks during refrigerated storage.

Several forms of interventions have thus been recommended to reduce the risks from* L. monocytogenes *in these products: (1) elimination or reduction of* L. monocytogenes* on the outside surface of frozen or fresh fish before filleting, (2) prevention of recontamination and growth of* L. monocytogenes* during all stages of processing, and (3) the inhibition of any possible survivors or recontamination during processing and distribution [44]. Numerous papers have been published on the inhibition of* L. monocytogenes* in cold smoked fish using physical interventions including gamma irradiation [47], X-ray irradiation [48], E-beam [49], high-pressure processing [50], chemical preservatives [51–53], natural antimicrobials [54, 55], and protective cultures [56, 57]. Extensive research has also been performed in the last decade on the application of antimicrobial packaging to specifically enhance the safety and extend the shelf life of fish and fish products. However, despite considerable efforts, this area of research remains challenging. This is primarily due to the intrinsic characteristics of fishery products themselves, namely, their almost neutral pH and presence of endogenous proteolytic enzymes [58], which can decrease the efficacy of acid antimicrobials and bacteriocins, respectively. Indeed, the effectiveness of salts of organic acids as antimicrobials is known to differ widely depending on the pH of the food matrix [16]. Moreover, in cold processed foods such as CSS, proteases can affect nisin stability [59].

Because of the aforementioned reasons, films and coatings have garnered more interest by virtue of their ability to not only provide a barrier against gases and moisture [60], but also act as carriers of antimicrobials. Alishahi and Aïder [61] pointed to the promising application of chitosan as an excellent antimicrobial, used stand alone or in combination, in herring, cod, cold smoked salmon, and trout. Reductions of the order of 1–3 log CFU/g of* L. monocytogenes*, following chitosan application on CSS, have been reported previously [32, 62]. Moreover, Gómez-Estaca et al. [63] coated cold smoked sardines with gelatin-based films (4%) enriched with oregano (1.25%) and rosemary extracts (20%) and showed that growth of TVC was retarded by 2 log and 2.5 log (respectively) compared to uncoated samples after 16 days of storage. Lu et al. [64] tested, in snakehead fish fillets, alginate coatings (20 mg/mL) with cinnamon EO (10 *μ*L/mL), EDTA (150 *μ*g/mL), and nisin (2000 IU/mL) alone and with their mixes against* Pseudomonas* spp., TVC, and psychrotrophic bacteria during storage at 4°C. The inhibitory effect of those antimicrobials on TVC followed the order: cinnamon + EDTA + nisin or cinnamon (5.5 log CFU/g) > nisin + EDTA (1.5 log CFU/g) compared to controls. Song et al. [65] reported that composite films of barley bran protein and gelatin containing grapefruit seed extract brought about a reduction of 0.5 log CFU/g of* E. coli* O157:H7 and* L. monocytogenes* on salmon after 15 days of storage at 4°C. The antilisterial effect of a calcium alginate coating incorporating oyster lysozyme in the presence or absence of nisin on the surface of smoked salmon was also investigated previously [66]. Although the coatings supplemented with nisin and lysozyme were able to delay or slow down the growth of* L. monocytogenes*, the treatment was not highly inhibitory. Taken together, these findings point to the highly variable antimicrobial efficacy of edible films and coatings (reductions of 0.5–5.5 log CFU/g), which is dependent on the type, concentration, and combination of antimicrobials as well as the test product of interest.

In our current study, the antimicrobial efficacy of cellulose-coated films and cellulose-based coatings incorporating Nis, SL, SD, PS, and SB in different concentrations and combinations was compared. Findings revealed that coatings incorporating nisin (25000 IU/mL), PS (0.3%), and SB (0.1%) were most effective and reduced the population of* L. monocytogenes* and anaerobic and aerobic spoilage flora by a maximum of 4.2, 4.8, and 4.9 log CFU/cm^2^_,_ respectively, after 4 weeks of refrigerated storage. The listeriostatic activity of nisin on cold smoked fish, at levels ranging from 100 IU/mL to 2000 IU/mL, alone or in combination with LAB protective cultures [67], or natural antimicrobials such as lyzozyme and polylysine [68], or chemical preservatives such as lactate or diacetate [69] has been demonstrated previously. Unlike our findings, Tang et al. [69] reported that a binary combination of Nisin and lactate had greatest antilisterial effectiveness. On the other hand, Wan Norhana et al. [70] showed that ternary combinations of nisin-PS-EDTA reduced the population of* L. monocytogenes* as well as psychrotrophic bacteria on vacuum-packaged shrimps by 1.3 and 4.0 log CFU/g, respectively. Neetoo et al. [31] also indicated that PS had considerable antilisterial activity when combined with nisin on CSS pâté and fillets. Sorbates have also been shown to kill or inhibit* L. monocytogenes* previously [25, 30, 71] and its activity can be further enhanced by the addition of nisin [72]. Other authors [72, 73] have similarly demonstrated the listeriostatic and listericidal ability of Nis + PS* in vitro* as well as on packaged beef kept at refrigeration temperature for up to 4 weeks.

Moreover, our data indicate that edible coatings containing nisin (25000 IU/mL) + PS (0.3%) or nisin (25000 IU/mL) + PS (0.3%) + SB (0.1%) reduced the population of* L. monocytogenes* and spoilage bacteria to a greater extent than their film counterparts. Recent studies have also highlighted the application of organic acids or their salts alone [74] or in combination with other hurdles such as CO_2_ [75] to delay the development of spoilage flora on salmon. Other overriding advantages of edible coatings are that they reduce packaging waste, they are environment-friendly, and they are low-priced [76, 77] when compared to films, which are synthetic packaging materials. Hence, edible coatings constitute an alternative, environmentally sustainable, and cost-effective technology [78] for the salmon industry. In the current work, the carrier of choice was cellulose, a widely available, low-cost, versatile polysaccharide biopolymer [77] and a compatible matrix for the embedded antimicrobials. Polysaccharide-based edible coatings are more popular than other hydrocolloids because they are generally transparent, cohesive, and homogeneous with adequate mechanical properties [60, 79].

Antimicrobials tested in this study are regarded as direct food additives and their application is thus limited by governmental legislation [16]. Sorbates may be directly added to food or incorporated into the packaging, at a level not exceeding 0.3% [23] while sodium benzoate is currently allowed at 0.1% [16]. Levels of PS (0.15 and 0.3%) and SB (0.05 and 0.1%) investigated in the current experiments were within legal limits of 0.3 and 0.1%, respectively. Presently, nisin is commercially added to smoked salmon in the United States (US) to control the growth of* L. monocytogenes*, although the maximum allowable level of this additive in smoked fish has not been stipulated [80]. The US Food and Drug Administration (FDA) has set a maximum limit of 10,000 IU/g for use of nisin in processed cheese although no such upper limit exists in Australia, France, or Great Britain [80, 81]. In the current study, the level of nisin incorporated into the films and coatings was 25000 IU/mL, translating to a maximum concentration of 2500 IU/g assuming complete leaching into the food product.

In recognition of the fact that CSS is (i) susceptible to postprocess surface contamination by* L. monocytogenes*, (ii) a refrigerated processed food of extended durability, and (iii) consumed without any heat-killing step, it is regarded as a high-risk product. This study reiterates the usefulness of antimicrobial packaging for cold smoked salmon, and it also underscores the effectiveness of cellulose-based coatings incorporating GRAS antimicrobials to control the development of pathogens and spoilage microbiota, thereby enhancing the microbiological safety and quality of this product.

## Figures and Tables

**Table 1:** Binary combinations of antimicrobials incorporated in films and edible coatings.

Antimicrobial films	Antimicrobial coatings
Control (plain LDPE films)	Plain MC/HPMC
Nis (25000 IU/mL)	Nis (25000 IU/mL)
Nis (25000 IU/mL) + SD (0.25%)	Nis (25000 IU/mL) + SD (0.25%)
Nis (25000 IU/mL) + SB (0.1%)	Nis (25000 IU/mL) + SB (0.1%)
Nis (25000 IU/mL) + SL (0.3%)	Nis (25000 IU/mL) + SL (0.3%)
Nis (25000 IU/mL) + PS (0.3%)	Nis (25000 IU/mL) + PS (0.3%)

**Table 2:** Binary and ternary combinations of antimicrobials incorporated in films and edible coatings.

Nisin (IU/mL)	PS (%)	SB (%)
0	0.0	0.0
25000	0.0	0.0
25000	0.0	0.05
25000	0.0	0.1
25000	0.15	0.0
25000	0.15	0.05
25000	0.15	0.1
25000	0.3	0.0
25000	0.3	0.05
25000	0.3	0.1

**Table 3:** Combinations of antimicrobials incorporated in films and coatings for the refrigerated storage study.

Nisin (IU/mL)	PS (%)	SB (%)
0	0	0
25000	0.3	0
25000	0.3	0.1
